# “I Can’t Move My Arms and Legs”: A Rare Cause of Hypokalemia-Induced Quadriparesis

**DOI:** 10.7759/cureus.16114

**Published:** 2021-07-02

**Authors:** Muhammad Atique Alam Khan, Artem Minalyan, Iqra Iqbal

**Affiliations:** 1 Internal Medicine, Abington Hospital–Jefferson Health, Abington, USA

**Keywords:** quadriparesis, hypokalemia, cocaine, toxicity, potassium

## Abstract

Hypokalemia is a relatively common electrolyte abnormality in hospitalized patients. Severe hypokalemia (<2.5 mEq/L) can lead to profound muscle weakness or paralysis, especially in the setting of acute onset of hypokalemia. Multiple mechanisms of hypokalemia have been described, such as decreased potassium intake, increased losses, and increased transcellular shift of potassium. Drugs can rarely cause hypokalemia by one of the above-mentioned mechanisms. Here, we report a case of cocaine use leading to severe hypokalemia manifesting as quadriparesis. The aggressive repletion of potassium led to a complete resolution of muscular weakness.

## Introduction

Cocaine is one of the most common causes of drug-related visits to the emergency departments in the United States [1]. Most of its important side effects are related to its hemodynamic effects (vasoconstriction leading to myocardial injury, stroke, seizures, etc.). These side effects are primarily mediated by the activation of the sympathetic nervous system. Severe hypokalemia has been reported to cause muscular weakness. Only a few cases have reported the association of cocaine ingestion leading to hypokalemia-induced muscular paralysis [2-5].

## Case presentation

**Figure 1:**
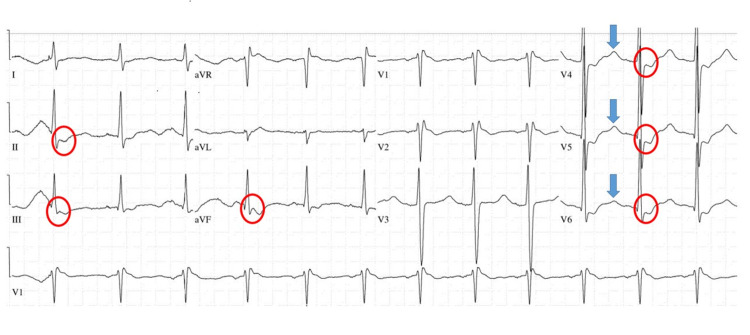
ECG demonstrating widespread ST depressions (red circles) with the presence of U waves (blue arrows). ECG: electrocardiogram

A 43-year-old Caucasian male was brought to the emergency department (ED) after he was found lying on the floor for more than 24 hours because of an inability to move his extremities due to profound weakness. Reportedly, the patient did not experience loss of consciousness, seizures, or focal weakness. His past medical history was most significant for anxiety, depression, and polysubstance abuse. Two days prior to the ED arrival, the patient took a bag of cocaine and six tablets of alprazolam. In the ED, his vital signs were unremarkable. On physical examination, profound motor weakness in all four extremities was noted (power: 1/5). Sensory examination and deep tendon reflexes were normal. The patient was awake and alert. On electrocardiogram (ECG), he was noted to have widespread ST depressions and U waves (Figure 1).

In his labs, he was noted to have a potassium level of 1.3 mEq/L, magnesium 2.7 mg/dL, bicarbonate 15 mEq/L, creatine kinase 911 U/L, blood urea nitrogen 16 mg/dL, and creatinine 1.27 mg/dL. Severe acute respiratory syndrome coronavirus 2 polymerase chain reaction was negative. Magnetic resonance imaging of the brain and cervical spine were unremarkable. The serum drug screen was also unremarkable. The urine drug screen was positive for benzodiazepines and cocaine. He was admitted to the medical intensive care unit for aggressive potassium and magnesium repletion and frequent electrolyte monitoring. Additional laboratory investigations were requested. Thyroid-stimulating hormone and cortisol levels were normal. The plasma renin-to-aldosterone ratio was slightly below normal. A spot urine potassium-to-creatinine ratio was not suggestive of urinary potassium wasting. After aggressive electrolyte repletion, the potassium started to improve (2.4 to 2.7 to 3.0). He initially got 60 mEq of potassium chloride in the emergency department. Later, in the intensive care unit, he was given additional doses of 40 mEq based on the repeated basic metabolic profile checks. His motor strength gradually improved. By day three of the hospitalization, no motor weakness was observed. The patient was discharged from the hospital and asked to have a close follow-up with his primary care physician. Unfortunately, the patient did not follow up with any healthcare providers after discharge.

## Discussion

Hypokalemia is a common electrolyte abnormality found in hospitalized patients. Potassium is mostly stored inside the cells, primarily in muscles. The Na-K-ATPase located in the cell membrane plays a key role in regulating the transcellular shift of potassium ions. The following three mechanisms can lead to hypokalemia: (1) decreased potassium intake; (2) increased potassium loss (perspiration, gastrointestinal, and urinary losses); and (3) increased cell entry [6]. Several factors can activate the Na-K-ATPase pump and, therefore, drive potassium intracellularly (causing hypokalemia). They include the effects of insulin and catecholamines. The latter mechanisms can be explained by the activation of beta-2 adrenergic receptors that can lead to the upregulation of Na-K-ATPase pump, Na-K-2Cl cotransporter, and by increasing insulin release [7]. The other proposed mechanisms of increased potassium entry into the cells include alkalosis, hypokalemic periodic paralysis (hereditary or acquired in the setting of hyperthyroidism), and increased uptake during the production of new blood cells, hypothermia, and drug intoxications (antipsychotics, chloroquine, etc.) [8].

In patients with established hypokalemia, obtaining a thorough history is crucial (use of drugs, diarrhea, vomiting, etc.). Laboratory evaluation includes the measurement of 24-hour urinary potassium excretion or spot urinary potassium-to-creatinine ratio. Of note, the urine potassium-to-creatinine ratio of more than 13 mEq/g of creatinine suggests urinary potassium wasting. In addition, the assessment of acid-base status should be determined in affected patients [9].

Cocaine is a well-known stimulant that acts as a triple reuptake inhibitor (serotonin-norepinephrine-dopamine) [10]. It is made from the leaves of the coca plant. Due to its sympathomimetic properties, cocaine toxicity can lead to side effects targeting multiple organ systems. They include cardiovascular (cardiac ischemia, myocardial infarction, arrhythmias, cardiomyopathy), nervous (seizures, stroke, movement disorders), respiratory (rhinitis, nasal septum perforation, hemoptysis, asthma exacerbation), gastrointestinal (xerostomia, ulcers, ischemic colitis, hepatitis), renal (acute kidney injury, hypertensive nephrosclerosis), endocrine (activation of the hypothalamic-pituitary-adrenal axis), and cutaneous (pseudovasculitic lesions when contaminated with levamisole) systems [11]. The ingestion of cocaine can cause pathological changes leading to hypokalemia via several mechanisms. As a powerful stimulant, cocaine exerts its effects via sympathetic pathways which can lead to the activation of the Na-K-ATPase pump leading to hypokalemia. Alternatively, cocaine can directly target potassium channels [12].

Cocaine can also cause rhabdomyolysis via cocaine-induced vasospasm [13]. In our patient, rhabdomyolysis was not severe enough to explain the degree of muscle weakness. This supports the conclusion that severe hypokalemia was the culprit for quadriparesis. In addition, rhabdomyolysis is known to cause hyperkalemia which was not evident in our patient. Although as a stimulant, cocaine is known to cause a myriad of clinical manifestations (mostly neuropsychiatric and cardiovascular), our patient did not develop the above-mentioned complications.

## Conclusions

In patients presenting with muscle weakness associated with hypokalemia, the use of recreational drugs should be considered. It is important to perform a comprehensive workup when evaluating patients, including serum and urinary drug panels. Notably, cocaine toxicity can present with an isolated muscular weakness in the setting of hypokalemia while other common side effects are absent. The repletion of potassium in affected patients can lead to a complete resolution of muscular weakness.
